# Effects of a Behavioral Program on Exercise Adherence and Exercise Self-Efficacy in Community-Dwelling Older Persons

**DOI:** 10.1155/2013/282315

**Published:** 2013-12-30

**Authors:** Azliyana Azizan, Maria Justine, Chua Siew Kuan

**Affiliations:** Faculty of Health Sciences, Puncak Alam Campus, Universiti Teknologi MARA (UiTM), 42300 Puncak Alam, Selangor, Malaysia

## Abstract

*Background. *This study determines the effects of a behavioral program on exercise adherence (step counts) and level of exercise self-efficacy (ESE) in community-dwelling older persons. *Methods. *Sixty-three participants (age = 63.8 ± 4.5 years) were enrolled in this controlled quasi-experimental study. They were divided into 3 groups: (1) EBG performed a 6-week exercise intervention followed by a 5-week behavioral program, (2) EG performed exercise intervention similar to EBG, and (3) control group (CG) did not receive any interventions. Step counts were measured based on the scores recorded by a pedometer while ESE was measured by a self-reported ESE scale. *Results. *Data analysis showed significant differences due to time effect (*F*(1,2) = 39.884, *P* < 0.01, and *η* = .399); time and group interactions (*F*(2,60) = 112.683, *P* < 0.01, and *η* = .790); and between-group effect (*F*(2,60) = 12.524, *P* < 0.01, and *η* = .295) for step counts. As for ESE, significant differences were also found for time effect (*F*(2,4) = 66.628, *P* < 0.05, and *η* = .526); time and group interactions (*F*(2,60) = 4.562, *P* = 0.014, and *η* = .132); and between-group effect (*F*(2,60) = 13.632, *P* < 0.05, and *η* = .312). EBG presented with significantly higher mean changes for both step counts and ESE compared to other groups (all *P* < 0.05). *Conclusion. *This study suggests that the addition of a behavioral program is superior as compared to exercising alone on increasing exercise adherence and level of self-efficacy in older persons.

## 1. Introduction

Purposeful exercise and increased participation in physical activity are among the most important components of lifestyle, especially for older persons [1]. Participation in these activities is considered an important behavior that may yield benefits especially on maintaining the physical and psychological well-being [2–5]. A published review on physical activity and aging highlighted that older persons who actively participated in a long term exercise program appear to have a good quality of life [6]. Regrettably, majority of old people around the world lead a sedentary lifestyle with very little knowledge regarding the importance of physical activity. As highlighted by Irvine et al. [7], almost 39% older persons do not meet recommended physical activity guidelines and 33% also reported no leisure-time physical activity. This meant that a portion of older people did not meet the recommended exercise program suggested by the American College of Sports Medicine [8]. An exercise program for older persons must include a multicomponent training, namely, endurance, flexibility, strengthening, and balance [8]. Older persons are encouraged to perform moderate intensity exercise 5 days per week with a minimum of 30 minutes per session. This recommendation aims to target all the major physiological systems that are functionally declining due to the aging process. For instance, a regular resistance training program may induce muscle hypertrophy by altering the expression of myosin heavy isoforms, [9], while aerobic exercise improves blood pressure and decreases the risk of coronary artery disease [10]. Balance training also helps in reducing fear of falls and improving dynamic balance [11]. Thus, it is clear that each training component may contribute to better physical health status.

One of the major issues related to participation in an exercise program is the compliance or adherence to such program that may be influenced or determined by numerous factors. A previous study has identified these determinants and grouped them into two main categories: either increase adherence to exercise (motivators) or decrease adherence to exercise (barriers) [12]. However, most studies consistently found that self-efficacy was the strongest predictor of exercise adherence [13–15]. Self-efficacy can be defined as perceived capability and confidence, specific to a particular domain of behavior [16]. In older persons, self-efficacy predicts exercise adherence [17], and this can be used to control and motivate them to engage in physical activity and exercise for as long as possible. Thus, this indicates a need to develop a theory-based physical activity and exercise intervention that can be linked to the concept of long term exercise adherence. This is vital since exercising has to be an ongoing activity in order to gain its immediate and long term positive effects.

Therefore, this study was carried out to develop interest and fostering motivation to help increase daily participation in physical activity among older persons living in the community through theory-based behavioral approaches. While the behavioral interventions have been revealed to provide positive outcomes in increasing level of physical activity [18], most, however, were applied and tested only for chronic illnesses such as depression [19], chronic low back pain [20], older persons with fear of falls [21], and diabetes type-2 [22]. There are a limited number of studies that have focused on the perception and behavior towards exercise, specifically among older persons residing in the community. Community-dwelling older persons are the main target against those institutionalized because they may differ in terms of their level of exercise adherence. One possible reason could be due to their other commitments, such as caring for grandchildren, running businesses, weekly meetings, and spiritual and voluntary activities. As such, this study was designed to determine the effects of a behavioral program intervention following a short term introduction to exercise program on exercise adherence and exercise self-efficacy. An exercise program was implemented prior to the initiation of the behavioral program to introduce the different components of exercise that are safe and effective for senior people. In this study, a pedometer was used to verify the step counts as a measure of level of physical activity [23, 24]. As is commonly known, a pedometer can detect steps accurately as an indication of volume of physical activity [25, 26]. In addition, the number or visual indicators on the pedometer can provide feedback in order to motivate people to increase their level of physical activity [26].

## 2. Methods

### 2.1. Study Design

A controlled quasi-experimental study was conducted over a period of 24 weeks. The participants were divided into three groups; (1) exercise and behavioral programs (EBG), (2) exercise program only (EG), and (3) control group (CG). The EBG received a six week exercise program adapted from a previous study [27], with increasing frequency and intensity. The exercise training was also added with a locomotor training such as dancing and tai chi movements. The termination of exercise was then followed by a behavioral program for another five weeks. The EG received the same exercise protocol as in group EBG for six weeks but without the behavioral program. While the control group (CG) was not given any interventions but instructed to continue their usual activities, the protocol for this study was approved by the Ethics Committee of the University. Each participant was assured of confidentiality and the option to withdraw at any time without penalty.

### 2.2. Study Setting and Participants

Ninety older persons aged above 60 years were recruited from three different villages, around Selangor Malaysia. Each village was allocated with only one type of intervention in order to avoid communications among the participants (Village 1 = EBG; Village 2 = EG; Village 3 = CG). Prior to the initiation of the intervention, all participants were screened for eligibility. Participants were included based on the following criteria; (1) aged 60 years old and above, (2) have no cognitive impairments (for those with MMSE score, <24 were excluded) [28], (3) independent in activities of daily living, (4) able to understand English/Malay, and (5) resided in the study setting. Subjects presented with chronic musculoskeletal problem, dementia or neurological disorders, severe hearing and visual impairment, and having an inability to walk were excluded from this study. Seventy-three older persons (EBG = 25, EG = 25, and CG = 23) who fulfilled the inclusion criteria were included in this study. At the end of the study, a few participants dropped out from the study, with remaining 18 in EBG, 23 in EG, and 22 in CG. The participants who were dropped out cited reasons such as no transportation to the study site, time constrains, and weather problems.

Participants from three different villages were chosen in order to reduce the effects of behavioral influence to other participants who may be motivated to perform the intervention that was not assigned to them. These villages were chosen based on their homogeneity in terms of level of education, ethnicity, previous nature of jobs, basic infrastructures (such as community halls and community clinic), and level of physical activities. At the time of the implementation of this study, none of the participants were carrying out any formal or informal exercise programs. As such, they were assumed to present with the same level of physical activity at baseline. We considered homogeneity among participants as an important factor to reduce the biasness in terms of baseline performance of physical activity. This is supported by Schöllgen et al. [29] that argued participants with significant differences in demographic status may have different levels of physical and psychological health especially in relation to the level of education and vice versa. Thus, this situation may affect their behavior towards exercise participation.

#### 2.2.1. The Exercise Program

This exercise program was designed based on the aspects of fitness for older persons and comprised of cardiovascular endurance, muscle strength, balance, flexibility, and locomotor training. The main target was to involve all major muscle groups of the body. The intensity and the volume of exercise were increased gradually to prevent any adverse effects of exercise. The exercise program consisted of 18 sessions with each lasted for about 60 minutes, carried out over a period of six weeks, three sessions a week. The exercises were conducted in sitting positions in the first two weeks (1st phase) then followed by standing positions in the last 4 weeks (2nd phase).

Each session consisted of 8 to 10 minutes of stretches for the major muscle group (pectoral, latissimus dorsi, cervical paravertebral, and posterior and anterior thigh muscles); 20 minutes of aerobic training; 20 minutes of strength training (with load determined by theraband and body weight); 10 minutes of locomotor, coordination, and agility and flexibility exercises. All sessions were conducted group based, supervised by the researcher and assisted by trained physiotherapists. The sessions were also incorporated with music, to keep the participants motivated, as suggested in a meta-analysis study [30]. A weekly meeting was held among the research members to ensure consistency of the sequence and pattern of the exercises for all participants.

#### 2.2.2. The Behavioral Program

The behavioral program was designed based on the “stages of change” model, which is one of the components in the “Transtheoretical model of behavior change” (TTM). This model was originally postulated by Prochaska et al. [31]. Several studies have found that the stages of change in TTM can be predictive of behavior change in older persons. For example, TTM was used for changing and implementing home safety modifications with community-dwelling older persons [32]. Another example includes the use of TTM for physical activity in older persons with type 2 diabetes and cardiovascular disease [33].

The aim of this behavioral program was to develop effective intervention to promote health behavior change, especially towards exercise participations. In essence, 5 modules were developed based on the 5 stages of change as postulated in TTM. For each stage of change, different behavioral strategies were implemented so that the older persons are motivated and may progress to the next stage.

The first stage is known as “precontemplation,” where, at this stage, older persons do not intend to take action within the next 6 months. They may be unaware of the benefits of changing behavior and thus may produce negative thoughts about it. They may focus more on the cons of behavior changes compared to the pros. Next, the second stage is “contemplation.” At this stage, older persons may intend to start healthy behavior in the future and may come to realize their problematic behavior. They may also consider the pros and cons of changing the behavior if it takes place, with equal emphasis placed on both. Even though they may recognize their problematic behavior, most may feel hesitant towards changing their behavior. This stage, however, will be followed by “preparation stage,” in which older persons are ready to take action within the next 30 days. They start to take small steps towards the behavior change, and they believe changing their behavior can lead to a healthier life. In the fourth stage, the “action stage,” older persons have recently changed their behavior and intend to keep moving forward with that behavior change. In this stage, older persons may modify their problematic behavior and may acquire new healthy behavior. The final part of the model will then be concluded by the “maintenance stage,” where older persons sustained their behavior change for a while or for the next 6 months, in order to go forward.

In this study, however, we assumed that all participants were in the precontemplation stage. This is to reduce overestimation, simply because of the complexity of the behavior itself towards exercise and physical activity [34]. The strategies used in this study were presented by the researcher and trained physiotherapists through powerpoint presentations, face-to-face interview, and counseling sessions.  Table 1 shows the protocol for the behavioral program used in this study, which was adapted from Jones and Rose [35] and Burbank et al. [36].

### 2.3. Study Outcome Measures

The primary outcome of this study is the level of physical activity also known as exercise adherence (steps count). The physical activity was measured objectively using a pedometer (Omron HJ-203; Omron Healthcare UK Ltd.). The pedometers were used for a period of 12 weeks, beginning at 12th week until 24th week after interventions. All records regarding the total daily step counts were documented in a pedometer sheet. The pedometer recorded step counts during participant's waking hours for seven days a week because it has a memory recall for up to seven days, allowing the research assistant to record any changes and recover the step counts in cases when participants failed to accurately record their step counts in the sheets [37]. The participants were asked to reach a target of 10,000 steps per day as recommended by the Ministry of Health, Malaysia, to stimulate people to take more frequent walks and carry out fitness activities [38]. Each participant wore the pedometer all day except during sleeping, bathing, and exercise program. The pedometer was positioned at the waist, in midline with the right middle thigh [39]. A research assistant who was blinded to the previous assessments and group allocation recorded participants' weekly step counts based on their own recording sheets.

The secondary outcome measure is the exercise self-efficacy which was measured by the exercise self-efficacy (ESE) scale [39]. The ESE scale is a self-reported questionnaire that contained nine items. The original English version of the ESE scale questionnaire was translated into Malay language (Bahasa Malaysia) based on the international guidelines [40]. The scales in the questionnaire measured the level of confidence on exercise performance started from 0 (not confident) to 5 (moderately confident) and up to 10 (very confident). Participants were instructed to rate their level of confidence on their ability to perform an exercise or walk for 20 minutes three times per week. The scale was scored by calculating the sum of the numerical ratings for each response which was divided by the number of responses.

### 2.4. Data Analysis

Changes in the number of steps and level of exercise self-efficacy were the main outcomes of this study. Results were reported as means (m) and standard deviations (SD). A one-way ANOVA and 3 × 2 Chi-square tests were used to determine the baseline demographic differences between groups. The analysis of covariance was conducted to determine the presence of confounding variables at baselines for all groups. The repeated measures analysis of variance (ANOVA) was used to determine whether there were time factor effects, group effects, and interaction between the two factors (group and time) for all groups. An alpha level of *P* < 0.05 was considered significant for all statistical tests.

## 3. Results

The final sample consisted of 63 participants (18 in EBG, 23 in EG, and 22 in CG group). The mean age of subjects was 63.8 (SD = 4.5 years). The mean height, body weight, and BMI were 1.55 (SD = 0.12 m), 63.53 kg (SD = 10.87 kg), and 26.42 kg/m^2^ (SD = 4.52 kg/m^2^), respectively. The baseline characteristics of study participants are shown in Table 2. There were no significant differences between the groups on demographic characteristics except for age variable, in which the significant difference was found between EBG and CG (*P* < 0.05).

The ANCOVA for age as the covariate was conducted for both number of steps and level of ESE at baseline. The results revealed that age was not a confounding factor for the number of steps (homogeneity of slopes, *F* = 0.131; *P* = 0.877. ANCOVA, *F* = 0.013; *P* = 0.910) and level of ESE (homogeneity of slopes, *F* = 3.042; *P* = 0.087. ANCOVA, *F* = 1.103; *P* = 0.298) at baseline. Therefore, further analysis using the repeated measures ANOVA was conducted to determine the effects of the intervention.

Table 3 shows that EBG attained the highest mean for number of steps at week 12 and week 24 compared to EG and CG. The repeated measures ANOVA showed significant differences in the number of steps at week 12 and up to 24 weeks due to the effect of time (*F*(1, 2) = 39.884, *P* < 0.01, *η* = .399); time and group interactions, (*F*(2, 60) = 112.683, *P* < 0.01, *η* = .790); and between-group effect (*F*(2, 60) = 12.524, *P* < 0.01, *η* = .295). The post hoc comparison using the Bonferroni test for mean difference between the three groups in the number of step counts showed a significant mean difference (EBG and EG = 43159.38; EBG and CG = 53725.53; EG and CG = 10586.15) with *P* value < 0.05.

Table 4 shows that the EBG scored the highest mean for ESE at week 24. Baseline level of ESE was not statistically significant different between the EBG, EG, and CG (*P* > 0.05). The repeated measures ANOVA showed significant differences in the level of ESE due to the effect of time (*F*(2, 4) = 66.628, *P* < 0.05, *η* = .526); time and group interactions (*F*(2, 60) = 4.562, *P* = 0.014, *η* = .132); and between-group effects (*F*(2, 60) = 13.632, *P* < 0.05, *η* = .312). However, the post hoc comparison using the Bonferroni test for mean difference between the three groups for ESE scores showed only significant difference between EBG and CG (10.90, *P* < 0.05). Further analysis showed that ESE score at week 12 was significantly different only between EBG and CG, while, at week 24, ESE differed significantly between EBG and EG as well as between EBG and CG (All, *P* = 0.01).

## 4. Discussion

The primary objective of this study was to determine the effects of a behavioral program following exercise participation on level of physical activity which was measured by the number of step counts recorded by a pedometer. The secondary objective was to determine the level of exercise self-efficacy (ESE) scores. The results obtained from this study are consistent with those of other meta-analysis studies of older persons that suggested behavior strategies were effective in changing behavior towards physical activity [41, 42]. There was a significant difference in the level of physical activity among the three groups. The EBG scored the highest mean average for the level of physical activity followed by the EG and CG.

The increased level of physical activity in the EBG indicates that the intervention may be effective in increasing participation in physical activity. However, both EG and CG recorded little difference in the number of steps. This is due to the fact that both did not receive any behavioral programs.

Participants in EBG showed a continuous increase in the number of steps which could largely be due to the agreement made by participants to comply with the behavioral contract, in which they were required to achieve a certain number of steps each week (e.g., 10,000 steps per day). This is advantageous as the pedometer can act as a visual feedback for older persons. The number of steps can be monitored personally at any time and users will be able to gauge the amount of steps needed to satisfy each individual's requirement. In addition, the behavioral contract could further motivate participants to adhere to the exercise activities. In contrast, without the behavioral contract, participants in the EG and CG lack the motivation needed to reach a higher number of steps.

As highlighted in a meta-analysis study, the use of a pedometer has a reasonable and constructive effect on the increase of physical activity across age groups, including older persons [43, 44]. This finding is in agreement with other reported studies that have used pedometer to measure level of physical activities based o steps count [45, 46]. However, we also argued that the pedometer may not be effective for older persons with slow walking speed and shuffling gait. As reported in a previous study, pedometers were less accurate in persons who “shuffle” their feet, such as elders in nursing home residents or those who lean on a walker [47]. Therefore, to minimize the bias and potential for ineffective results, we only include participants with reasonable walking pattern or those without shuffling gait.

In addition, the findings of this current study suggest that an exercise program combined with behavioral program is effective not only in increasing walking distance but also in improving older person belief and knowledge about the importance of exercising and physical activity. This can be seen among the participants in EBG who received additional behavioral program, as they were introduced with specific modelling, goal-setting, self-monitoring, and performance feedback. Thus, this supports the importance of the provisions of knowledge and skills related to exercise and physical activity in order to facilitate a behaviour change.

Generally speaking, older persons with a high level of self-efficacy may have better participation and engagement in a long term physical activity and exercise. In this study, participants who received exercise and behavioral program (EBG) have high levels of exercise self-efficacy, specifically at the 24th week. Their level of confidence in performing an exercise kept increasing as they want to achieve their short term and long term goals. Participants revealed that their goals are most useful when they are specific, realistic, and important for them. When setting their own specific goal, (e.g., walking 3 km for this week and increase 2 km for the following weeks), researchers continuously reminded participants to see and review the list of goals regularly. This might influence the inner motivation needed in order for participants to keep performing physical activity or exercise. This view is consistent with a previous study that had found a strong association between exercise self-efficacy and level of exercise participation among older African-Americans [48]. Self-efficacy has the benefit of significantly predicting exercise behavior at follow-up when biological and behavioral influences were controlled [48]. Therefore, in this behavioral program, we included the module of goal setting, the barriers and motivators to exercising, and other parameters as well as a module regarding the risk and benefits of exercise.

The behavioral program also allows the researchers to promote and support exercise as a constructive means of prevention while incorporating it into general health promotion activities. Participants in the EBP were also found to have significantly increased level of self-confidence while performing exercise compared to both EG and CG due to the power of leadership. In fact, the participants in EBG formed their own exercise group after the termination of the behavioral program without any support from the researchers. Exercises were conducted and performed based on the recommendations of the behavioral manual. Each participant showed strong commitment and cooperation especially in reminding each other regarding the type, frequency, and time to exercise. The role of group support may indicate the importance of the partnership factor and can be applied in any kind of situations. For instance, participants in the group can remind their partners of their strengths and how even seemingly small contributions can help in increasing the belief of performing an exercise.

As revealed in the results of this current study, participants in EG and CG also showed an increase in their belief of exercising based on the exercise self-efficacy score. Nonetheless, the ESE scale is a self-reported questionnaire, and this might contribute to bias among the participants. Affuso et al. [49] suggested that self-reported behavioral questionnaires may result in subjects intentionally providing inaccurate answers during interview due to pressure or socially acceptable manner. Other studies suggested that the influence on cognitive parts in the behavioral program [50–52].

In this study, the interventions were conducted in a group-based setting. We believe that a group-based intervention may yield better results compared to individualized program as it may enhance social support and increase skills in building relationship between members of the group. According to Bartels [53], older persons can gain benefit more from group interaction, when groups are composed of other older persons. However, an individualized program may be more effective for special cases such as older persons with depression [54]. Nevertheless, future study should attempt to establish older person's preference on the type of intervention as each person may have different levels of motivation.

Several limitations were noted in this study, particularly in research involving quasi-experimental design. This design lacks randomization to intervention groups, which characterizes true experiments. Furthermore, this study only focused on Malay ethnic residing in the community that may lead to bias to other races and setting. Future studies should involve diverse ethnicity due to the differences in the cultural belief and behavior.

Lastly, the findings and recommendations from this study are beneficial and crucial as it may increase exercise adherence and self-efficacy among community-dwelling elderly or specifically for those having barriers towards exercise participations. This study has established the combination of physical and psychological approaches with the intention to maximize older persons' psychological resources, namely, exercise adherence and exercise self-efficacy. Successful ageing is not only about the maintenance of health and exercising regularly but also about positive behavior and attitudes towards exercise participation. These lifestyles changes in older people may have beneficial effects on health and longevity, as well as on the enhancement of quality of life.

## 5. Conclusion

As a conclusion, this study suggests that the behavioral program with a pedometer-based feedback may be an effective intervention especially if the aim is to increase the level of physical activity. Interventions such as a combination of goal setting, contract, feedback, consequences, self-monitoring, and prompts in addition to a multicomponent exercise program may be beneficial for older persons residing in the community.

## Figures and Tables

**Table 1:** The behavioral intervention protocol.

Activities	
*Week 1. Precontemplation *	
Goal: increase awareness of the need for change Change process: consciousness raising, dramatic relief, and environmental reevaluation	
Strategies: (1) encourage thinking about change	
(2) discuss feelings and perceptions of exercise	
(3) give personalized feedback about the risk of sedentary lifestyle and low exercise tolerance	
(4) personalize information about the benefits of initiating an exercise program and how inactivity affects role implementation and social activities	

*Week 2. Contemplation *	
Goal: motivate and increase confidence in the ability to change	
Change process: consciousness raising, self-reevaluation, social liberation, self-liberation, and help participant to set realistic expectations reinforced by small success	
Strategies: (1) continue explaining regarding personal risk and benefits about exercising	
(2) decrease the barrier to exercise	
(3) help them to make a definite commitment to change and goal setting	
(i) Barriers and motivators worksheet was provided	
(ii) Encourage participants to replace unhelpful thoughts with more productive thought into a “Positive Self Talk” sessions.	

*Week 3. Preparation *	
Goal: negotiate a plan for exercise	
Change process: self-reevaluation, helping relationship, self-liberation, and help participant to set realistic expectations reinforced by small success	
Strategies: (1) create a new self-image as an exerciser	
(2) make a public commitment to exercise	
(3) identify alternative for an exercise at home, individually or in a group.	
(4) facilitate exercise efficacy involving support from others such as spouse and children	
(5) encourage a component of behavioral change where they can experience success	
(i) Barriers and motivators worksheet was provided	
(ii) Use “Behavioral Contract and Presentation entitled “How to handle stress”” by the researcher.	

*Week. 4 Action *	
Goal: reaffirm commitment to exercise and implement the plan	
Change process: reinforcement management, helping relationship, counter-conditioning, and stimulus control	
Strategies: (1) talk on positive reinforcement with rewards/incentives and “role model” for the other group	
(2) mobilizing social support to participate in the process or reinforce the process	
(3) introduce additional exercise and correct techniques during exercising through behavioral manual given to each of the participants	
(4) exercise log book and a group task	

*Week 5. Maintenance *	
Goal: problem solving	
Change process: counter-conditioning, helping relationship, and reinforcement management	
Strategies: (1) plan for resisting temptation to skip exercise sessions	
(2) join an exercise group	
(3) a meaningful reward for the successful exerciser	
(4) behavioral manual	

(i) All strategies were done through powerpoint presentations, face-to-face interview, and counseling, supervised by the researcher and trained physiotherapists.	
(ii) Every Monday and Wednesday (30 min) for each week.	

**Table 2:** Characteristics of study participants (*n* = 63) at baseline.

Characteristics	EBG Group (*n* = 18)		EG Group (*n* = 23)		CG Group (*n* = 22)		Sig
	*n* (%)	m ± SD	*n* (%)	m ± SD	*n* (%)	m ± SD	
Age (years)		66.1 (6.21)		63.5 (3.39)		62.3 (3.07)	0.025* (EBG-CG*)
Gender							
Female	8 (44.4)		12 (54.5)		15 (65.2)		0.411
Male	10 (55.6)		10 (45.5)		8 (38.4)		
Body weight (kg)		59.82 (3.33)		65.89 (2.16)		64.187 (1.66)	0.202
Height (m)		1.53 (0.03)		1.56 (0.02)		1.57 (0.02)	0.521
BMI (kg/m^2^)		25.83 (1.58)		27.05 (0.65)		26.27 (0.761)	0.692

Note. *Significant at *P* < 0.05.

**Table 3:** Number of step counts for three groups across two point intervals.

Time period	EBG group			EG group			CG group			Sig
	*n*	Mean	SD	*n*	Mean	SD	*n*	Mean	SD	
Baseline	18	1491.28	220.134	23	1737.18	225.117	22	1627.09	183.688	0.724
12th week	18	48495.19	7884.38	23	12708.10	11144.67	22	6905.41	1609.12	0.00**
24th week	18	73883.75	15612.10	23	23352.06	27460.122	22	7982.46	3626.373	0.00**

Note. **P < 0.01.

**Table 4:** Exercise self-efficacy scores for three groups across three time periods.

Time period	EBG group			EG group			CG group			Sig
	*n*	Mean	SD	*n*	Mean	SD	*n*	Mean	SD	
Baseline	18	35.00	14.65	23	39.09	19.00	22	38.70	16.31	0.698
Week 12	18	53.33	10.29	23	48.86	11.332	22	41.30	14.24	0.009*
Week 24	18	73.83	8.459	23	52.73	13.950	22	49.48	16.35	0.000*

Note. *Significant at *P* < 0.05.
